# Mapping research trends in shared decision-making for type 2 diabetes mellitus: a bibliometric study

**DOI:** 10.3389/frhs.2026.1799382

**Published:** 2026-05-28

**Authors:** Carla Salgado-Castillo, Christian García-Mera, María José Hernández-Leal, Carlos Manterola

**Affiliations:** 1 PhD Program in Medical Sciences, Universidad de La Frontera, Temuco, Chile; 2 Systematic Reviews and Documentary Research Group, School of Medicine, Universidad del Azuay, Cuenca, Ecuador; 3 University of Navarra, School of Nursing, Department of Community, Maternity and Pediatric Nursing, Campus Universitario, Pamplona, Spain; 4 IdiSNA Navarra Institute for Health Research, Pamplona, Spain; 5 Department of Surgery, Center of Morphological and Surgical Studies (CEMyQ), Universidad de La Frontera, Temuco, Chile

**Keywords:** bibliometric analysis, bibliometrix, diabetes, patient-centered care, shared decision-making

## Abstract

**Background:**

Type 2 Diabetes Mellitus (T2DM) requires continuous and complex decision-making, including treatment initiation, therapy intensification, and lifestyle considerations. Shared Decision-Making (SDM) is recommended to align clinical evidence with patients’ values and contexts. However, scientific production on SDM in T2DM is fragmented across disciplines, making it challenging to identify conceptual evolution, intellectual influences, and implementation gaps.

**Methods:**

We conducted a bibliometric analysis of publications on SDM in T2DM, indexed in Web of Science, Scopus, and PubMed, up to December 31, 2024. Records were deduplicated and screened; studies mentioning SDM only superficially or focusing on adjacent constructs (e.g., general patient education without explicit SDM analysis) were excluded. Data were analyzed using Bibliometrix (R) to explore productivity, collaboration, and thematic structures. Social networks (countries, institutions, authors) and intellectual networks (co-citation) were constructed, and a thematic map was generated using Callon's centrality and density.

**Results:**

A total of 272 documents (2000–2024) authored by 1,307 researchers from 412 institutions in 31 countries were included in this analysis. Publications increased at an annual rate of 14.5% and peaked in 2023. The United States led in output and collaborations, followed by the United Kingdom and the Netherlands. Mayo Clinic was the most productive institution (82 articles), while Montori VM was the most influential author. Core journals included *Patient Education and Counseling* and *BMC Health Services Research*. Core themes included patient decision aids and adherence, while emerging topics involved minimally disruptive medicine. Collaboration is concentrated in the Global North, with biases toward Anglo-European settings.

**Conclusion:**

Research on SDM in T2DM is expanding but remains focused on Anglo-European contexts, with limited participation from the Global South. Strengthening global collaboration and addressing underrepresented perspectives are crucial to developing more inclusive, patient-centered SDM models for diabetes care.

## 1 Introduction

Type 2 Diabetes Mellitus (T2DM) is a disease that requires ongoing, complex decisions — including medication initiation/intensification, insulin initiation, and preferences regarding adverse events, cost, and lifestyle — in which patients’ values and circumstances are decisive (1, 2). Shared Decision-Making (SDM), particularly when supported by patient decision aids, is recommended to bridge the gap between clinical evidence and personal choices. A landmark Cochrane review concluded that decision aids improve knowledge, risk perceptions, value clarity, and active participation in decision making, while raising the likelihood of informed, values-congruent choices (1). Organizations such as the American Diabetes Association further endorse this approach, noting that SDM has been shown to improve glycemic control (glycated hemoglobin, HbA1c) (2, 3).

Although the literature on SDM in T2DM spans multiple disciplines (medicine, nursing, psychology, and health informatics), it remains heterogeneous and fragmented (4), hindering the identification of conceptual evolution, key intellectual influences, and implementation gaps. Previous bibliometric studies have mapped general trends in SDM research (5, 6) or examined diabetes research without expressly addressing SDM as a central construct (7–10). Therefore, this study aims to map the global research landscape of SDM in T2DM through analyzing its productivity, collaboration networks, intellectual structure, and thematic evolution.

## 2 Methods

### 2.1 Search strategy and data sources

On March 10, 2025, we conducted a comprehensive search in Web of Science (WoS), Scopus, and PubMed covering literature published up to December 31, 2024. We limited the search to documents in the “articles”, “reviews”, “proceeding papers”, and “abstracts” categories that focused on SDM and type 2 diabetes mellitus (T2DM). We used English and Spanish search strings, but did not restrict retrieval by publication language. As an example, the search strategy used in Web of Science (Topic field, TS) was as follows:

TS = (((“shared decision making” OR “shared decision-making” OR “patient-centered decision making” OR “patient centred decision making” OR “informed decision making” OR “informed decision-making” OR “participatory decision making” OR “participatory decision-making” OR “joint decision making” OR “joint decision-making” OR “toma de decisiones compartidas” OR “toma de decisiones compartida” OR “herramienta decisional” OR “decisiones informadas” OR “participacion en la toma de decisiones”) OR [“patient decision aid*” OR “decision aid*” NEAR/3 (patient* OR shared OR participat*)]) AND [(“type 2 diabetes” OR “type 2 diabetes mellitus” OR “type II diabetes” OR “type II diabetes mellitus” OR “diabetes mellitus type 2” OR “diabetes mellitus type II” OR “T2DM” OR “T2D” OR “diabetes mellitus tipo 2” OR “diabetes mellitus tipo II”)] NOT (“software defined memory” OR “semantic data model*” OR “SDN” OR “SDM-IO”) NOT (“physician-only” OR “clinician tool*” OR “doctor decision*” OR “provider tool*”))

The complete search strategies adapted for each database are provided in Supplementary File 1.

### 2.2 Inclusion and exclusion criteria

We included documents that explicitly addressed SDM as a central concept, process, outcome or framework in the context of T2DM (e.g., development/evaluation of SDM interventions, assessment of patient/provider perceptions of SDM, or empirical analysis of SDM implementation).

We excluded documents in which SDM was mentioned only incidentally (e.g, in background or discussion without empirical data, intervention or analysis), or where the main focus was on adjacent constructs (health literacy, diabetes education, or patient empowerment) without direct examination of SDM.

### 2.3 Screening and data processing

Two review authors independently screened the titles and abstracts of all records identified, followed by full-text assessment of potentially eligible records. Disagreements were resolved through consensus among the research team. Inter-rater agreement was assessed using Cohen's kappa statistic, yielding a value of 0.80.

Records from the three sources were de-duplicated (primary key: DOI; secondary key: exact title and year). Author affiliations were standardized (e.g., Leiden University with Leiden University Medical Center; University of Illinois Chicago with its hospital variants) to minimize institutional fragmentation.

### 2.4 Data analysis

Analyses were conducted in R using the bibliometrix package (10) (including its Biblioshiny interface) and complementary packages: ggraph (11), igraph (12), rnaturalearth (13), ggplot2 (14).

#### 2.4.1 Bibliometric indicators and descriptive analysis

We calculated standard bibliometric indicators, including the number of publications per year, annual growth rate, average citations per document, most productive authors, institutions, countries, and journals. Journal dispersion was evaluated according to Bradford's law (15) using the Bibliometrix implementation to identify the core (Zone 1) and peripheral zones, along with the proportion of papers in each zone. Global citation counts were based on the combined dataset from all three databases.

#### 2.4.2 Collaboration and social network analysis

Country- and institution-level co-authorship networks were constructed, with edge weights representing the number of co-authored papers. We computed network centrality indicators (degree/strength, PageRank, betweenness centrality to identify key intermediaries, and closeness centrality) and detected communities using the Louvain algorithm (16). International collaboration was quantified as the percentage of multi-country publications (MCP%).

#### 2.4.3 Intellectual structure (co-citation analysis)

We constructed a cited-reference co-citation network and computed centralities to identify influential and bridging works, using only records identified from the Web of Science database (89% of the data), since PubMed and specific records in Scopus did not provide full cited references in the exported format. However, global citation analysis and productivity incorporated results from the full combined dataset.

#### 2.4.4 Conceptual structure and thematic mapping

Author keywords were pre-processed through (1) lower-casing, (2) synonym mapping (user-defined), (3) removal of generic stop terms (e.g., male/female/adult/middle-aged/aged), and (4) standardization of hyphenated and spacing variants (e.g., patient-centered/patient centred, self-management/self management). To avoid dominance in visualizations, the anchor terms “shared decision-making” and “diabetes mellitus” were suppressed. A co-word matrix was constructed and normalized using association strength. Clusters were identified with the Louvain algorithm, and each cluster was positioned on a thematic map according to Callon's centrality (*x*-axis) and density (*y*-axis). Labels for each theme display the 2–3 highest-frequency terms (17, 18).

## 3 Results

**Figure 1:**
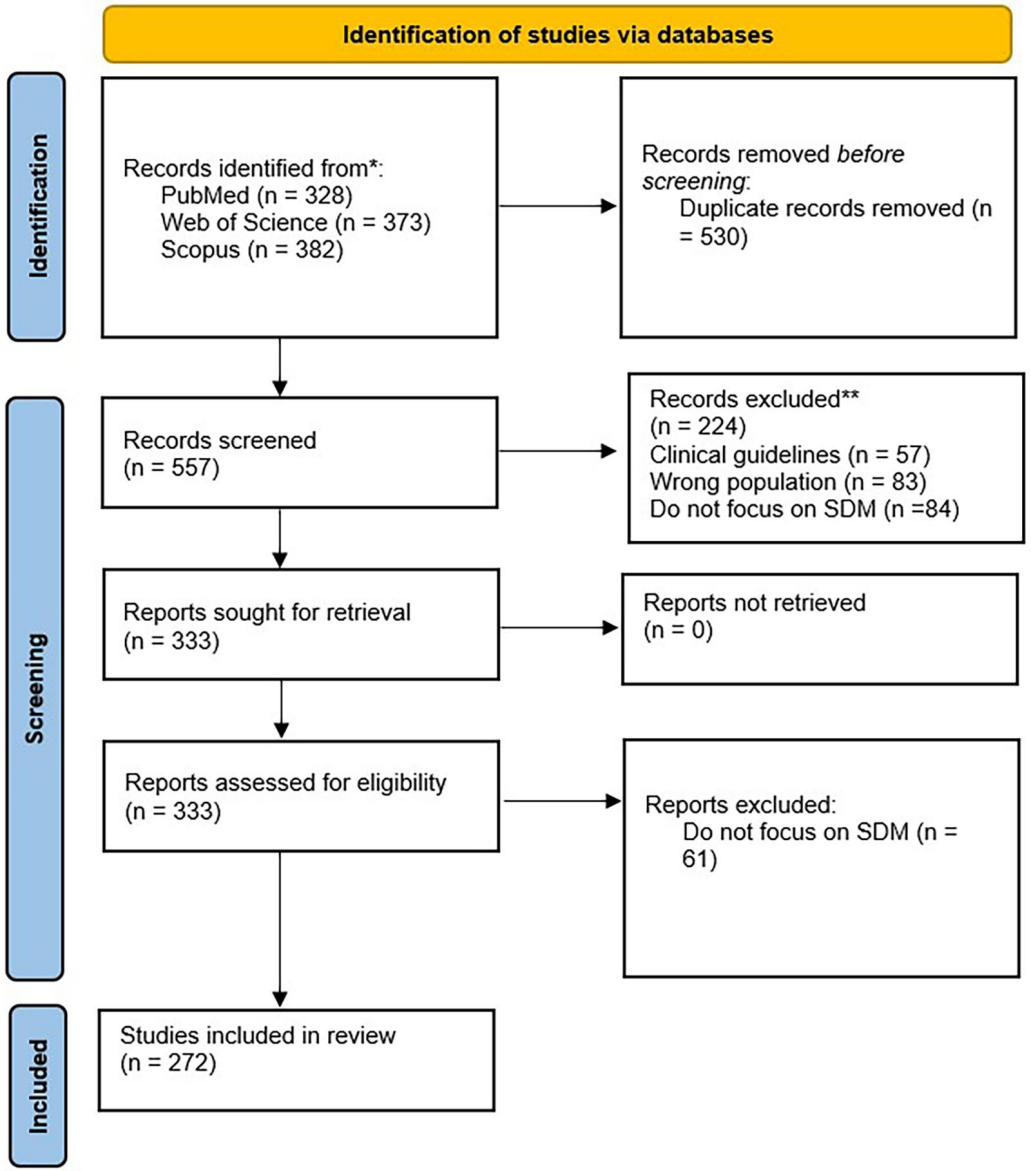
PRISMA-style flow diagram of the study selection process for the bibliometric analysis of shared decision-making in type 2 diabetes mellitus (2000–2024). Page et al. (62)

**Figure 2:**
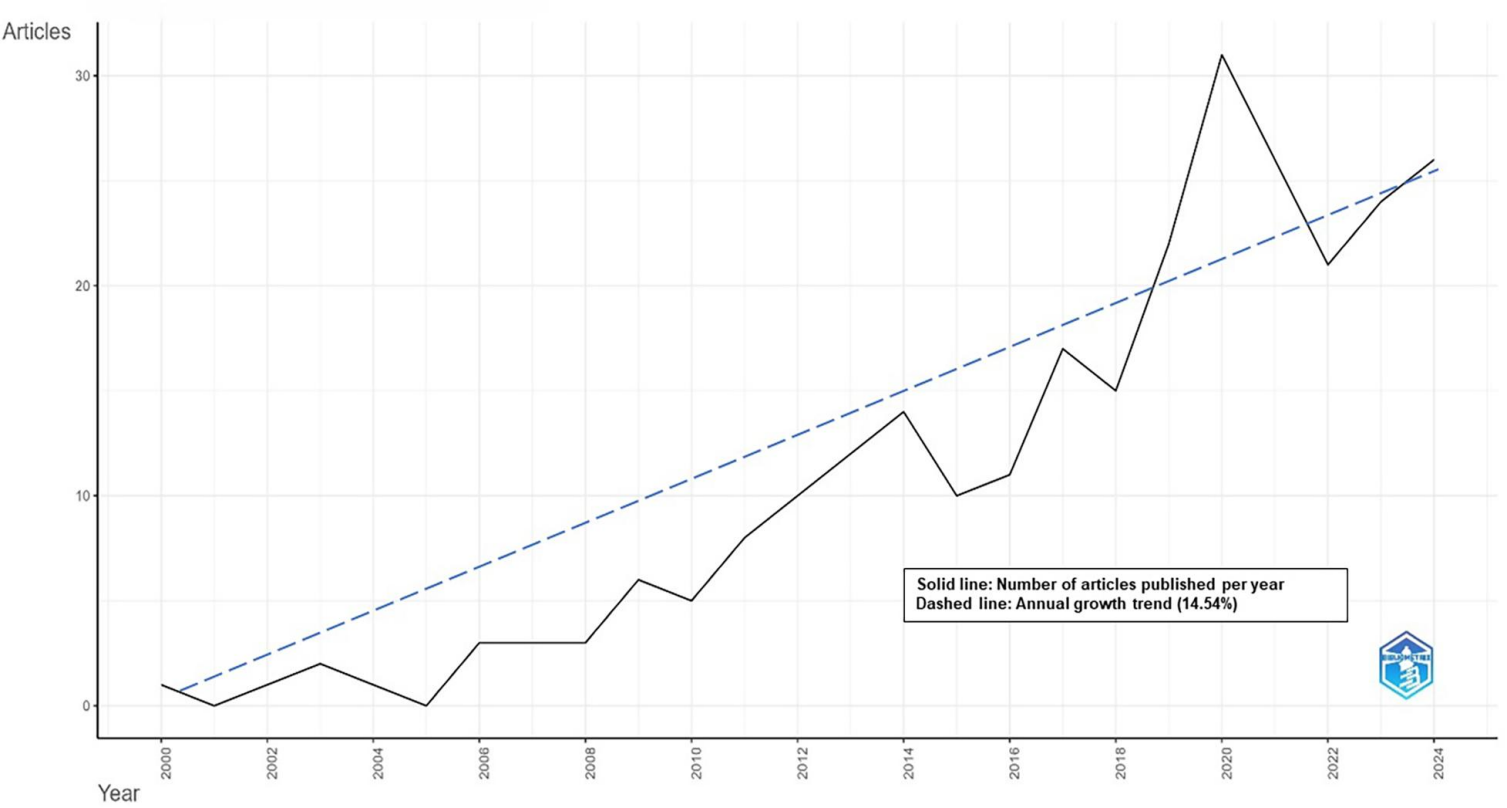
Annual scientific production of publications on shared decisioN,making in type 2 diabetes mellitus from 2000 to 2024 (*N* = 272 documents). The solid line represents the number of articles published each year. The dashed line indicates the annual growth trend of 14.54%.

Figure 1 illustrates the study selection process. A total of 272 articles, published between 2000 and 2024, were obtained, authored by 1,307 researchers from 412 institutions across 31 countries. Table 1 gives a comprehensive review of all basic information, while Supplementary File 1 presents a list of the core document collection. Publications grew at an annual rate of 14.54%, with 2023 being the year with the highest number of publications (*N* = 24) (Figure 2).

**Table 1:** Bibliometric analysis: main overview.

Description	Results (n)
Documents	272
Period	2000–2024
Sources (Journals, Books, etc.)	131
Annual Growth Rate %	14.54
Document Average Age	7.18
Average citations per document	22.87
References	7,266
Citations	6,221
Keywords Plus (ID)	652
Author's Keywords (DE)	656
Authors	1,307
Single-authored Documents	5
Co-Authors per Document	6.36
International co-authorships %	24.63
Article	229
Review	31
Abstracts/Proceedings Paper	12

### 3.1 Analysis of countries and institutions

**Figure 3:**
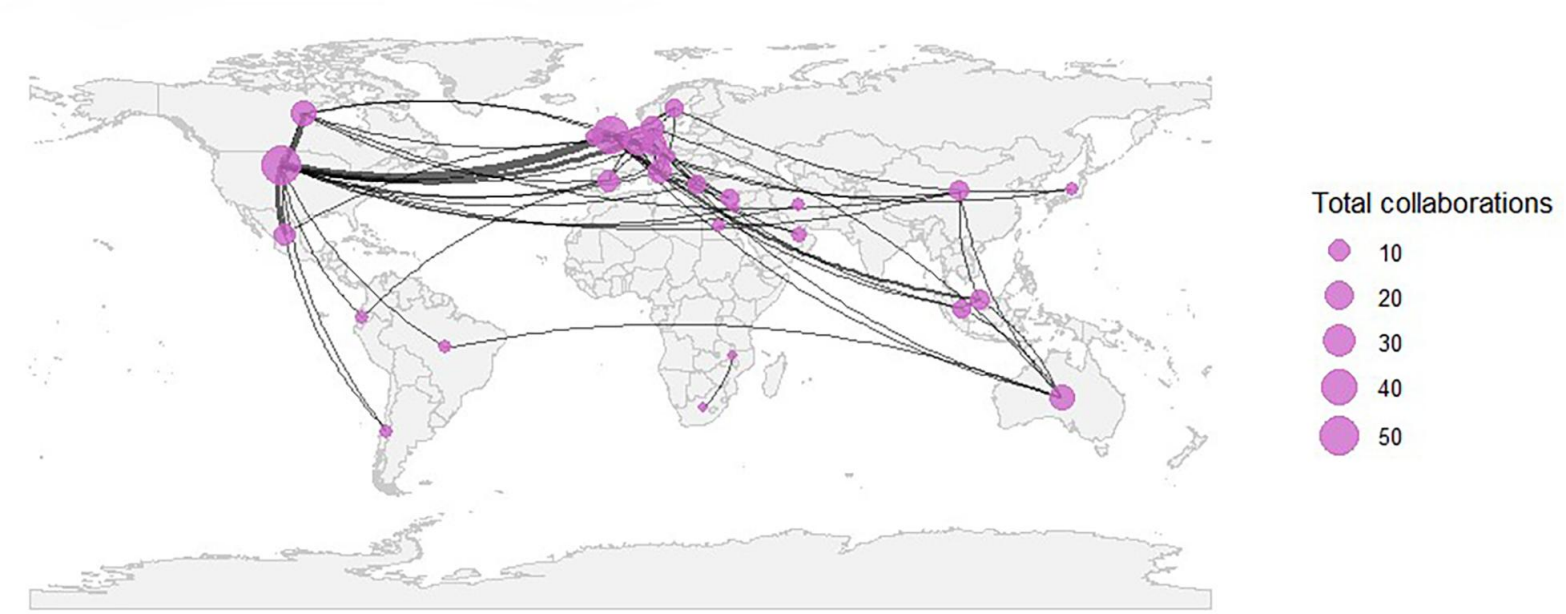
**Country,level collaboration network on shared decision, making in type 2 diabetes mellitus (2000−2024).** Node size is proportional to the number of publications per country; edge thickness represents the number of collaborative papers between countries.

Across 352 country-level contributions, 47.2% were part of multi-country collaborations (MCPs). The United States leads with 118 contributions (MCP ratio 0.31), followed by the United Kingdom with 32 (MCP 0.72), the Netherlands with 30 (MCP 0.33) (Table 2). The country-level collaboration network showed modest community structure (Louvain modularity Q = 0.218), with the United States and United Kingdom acting as central brokers (betweenness centrality: 68.5 and 61.0, respectively) (Figure 3).

**Table 2:** Top 10 countries in shared-decision making in T2DM.

Country	SCP (n)	MCP (n)	Total (n)
United States	82	36	118
United Kingdom	9	23	32
Netherlands	20	10	30
Germany	16	6	22
Canada	10	9	19
Australia	9	9	18
China	10	5	15
Denmark	4	8	12
Malaysia	7	4	11
France	2	8	10

MCP, multiple-country publications; SCP, single-country publications; T2DM, type 2 diabetes mellitus.

**Figure 4:**
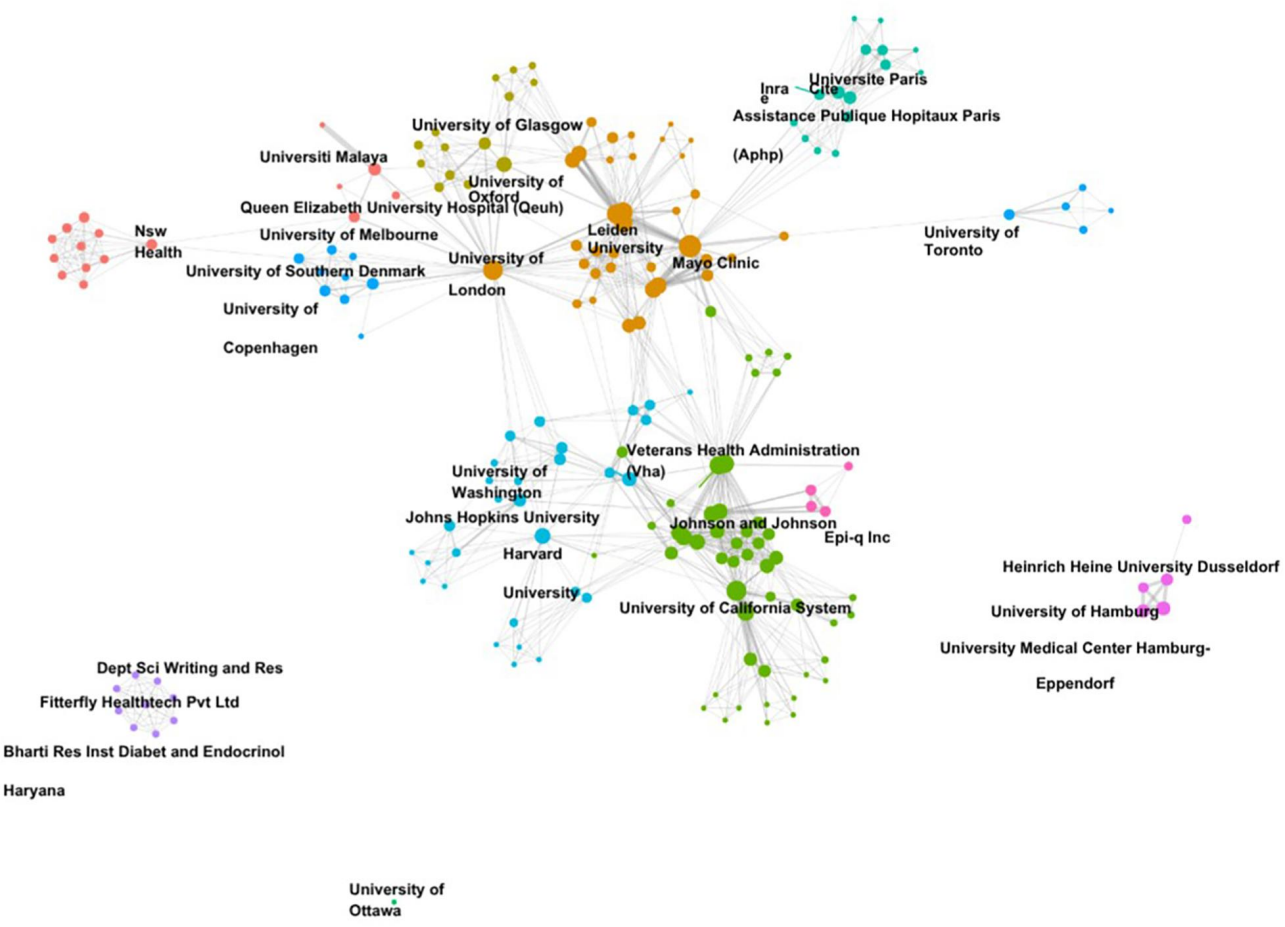
**Institutional collaboration network on shared decision, making in type 2 diabetes mellitus (382 institutions).** Node size is proportional to the number of publications; edge thickness represents the strength of co-authorship. Colors denote communities identified by the Louvain algorithm.

At the institutional level, Mayo Clinic led with 82 publications, followed by Harvard University (34) and Maastricht University (26) (Table 3). The institutional network revealed a core–periphery structure with dense communities in North America and Europe (Figure 4).

**Table 3:** Top 10 institutions in shared-decision making in T2DM.

Affiliation	Articles (n)
Mayo Clinic	82
Harvard University	34
Maastricht University (MUMC)	26
University of Chicago	24
University of California System	23
Universiti Malaya	20
University of Toronto	18
Leiden University	17
University of Washington	16
Johns Hopkins University	15

### 3.2 Analysis of journals

Publications were distributed across 131 journals (272 articles). The core zone (Zone 1, Bradford's Law) comprised 11 journals accounting for 33.2% of papers. The core outlets are listed in Table 4, where *BMC Health Services Research* and *Patient Education and Counseling* lead with 12 articles each.

**Table 4:** Top 10 journals publishing shared-decision making in T2DM.

Sources	Articles (n)
*Patient Education and Counseling*	12
*BMC Health Services Research*	12
*BMJ Open*	9
*Health Expectations*	9
*Diabetic Medicine*	8
*Journal of General Internal Medicine*	8
*Patient Preference and Adherence*	8
*BMC Family Practice*	7
*Canadian Journal of Diabetes*	7
*Diabetologia*	5

### 3.3 Analysis of authors and references

**Figure 5:**
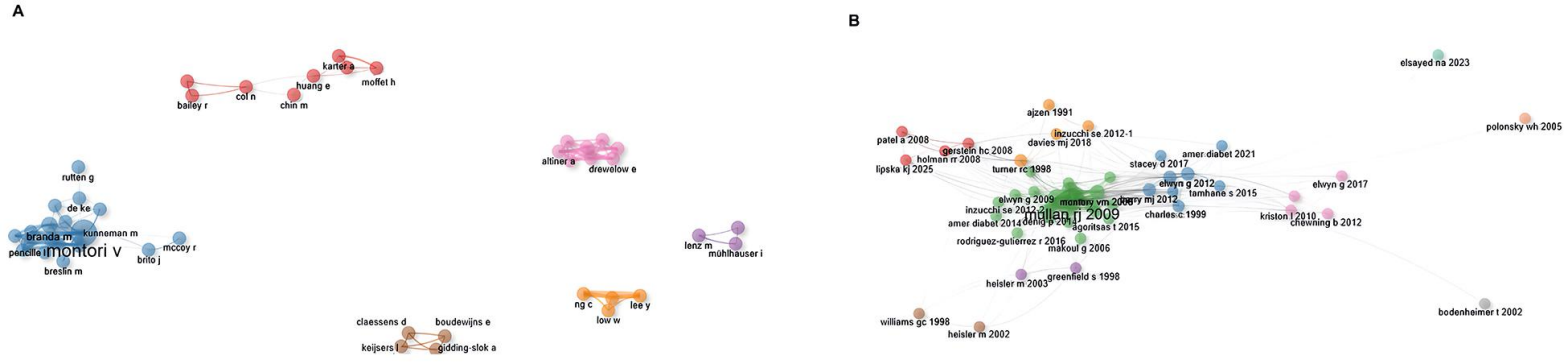
**(A) author collaboration network.** Node size is proportional to the number of publications per author; edge thickness reflects co-authorship frequency. **(B) CO, Citation network of cited references based on the Web of Science subset (50 nodes).** Node size represents citation frequency; colors indicate communities detected by the Louvain algorithm.

The top 10 authors and most globally cited references (by total citations) are presented in Table 5. Montori V.M ranks as the top author in the field, with 25 articles, and Arora N., 2000 (19). is the most-cited reference worldwide (*N* = 429). By centrality analysis, Montori V.M is also the most influential author (highest PageRank: 0.0566) and acts as the principal broker (highest betweenness: 54.562). Other groups show very high cohesion locally but minimal external connectivity (Figure 5A) Supplementary material.

**Table 5:** Most productive authors and most globally cited references.

Rank	Authors	Articles (n)	References	Count
1	Montori V	25	Arora N, 2000, MedCare (19)	429
2	Ng C	11	Montori V, 2006, Health Expect (56)	297
3	Lee Y	10	Murphy H, 2008, BMJ (57)	262
4	Branda M	8	Cummings D, 2016, Diabetologia (58)	228
5	Shah N	7	Mullan R, 2009,Arch Intern Med (20)	227
6	Lee P	6	Weymiller, 2007, Arch Intern Med (21)	215
7	Rutten G	6	Karter A, 2010, Diabetes Care (33)	194
8	Denig P	5	Parchman m, 2010, Ann Fam Med (59)	186
9	Altiner A	5	Breslin M, 2008, Patient Educ Couns (60)	126
10	Drewelow E	5	Montori V, 2007, Plos Med (61)	119

The cited-reference co-citation map from records obtained from Web of Science (50 nodes; 10 communities) **-**Figure 5B**-** reveals a dominant core cluster centered on Mullan, R.J. (2009) (20), Weymiller, A.J. (2007) (21), Mann, D.M. (2010) (22), Stacey, D. (2017) (24), and Branda (2013) (23), which are the most influential references by PageRank (Supplementary Material). Bridging analysis showsthat Charles (1997) (25), Barry (2012) (26), Elwyn (2012) (27), and Turner (1998) (28) have the highest betweenness. The remaining communities are compact, thematically cohesive satellites with limited cross-links to the core, consistent with a core–periphery intellectual structure.

### 3.4 Analysis of keywords

**Figure 6:**
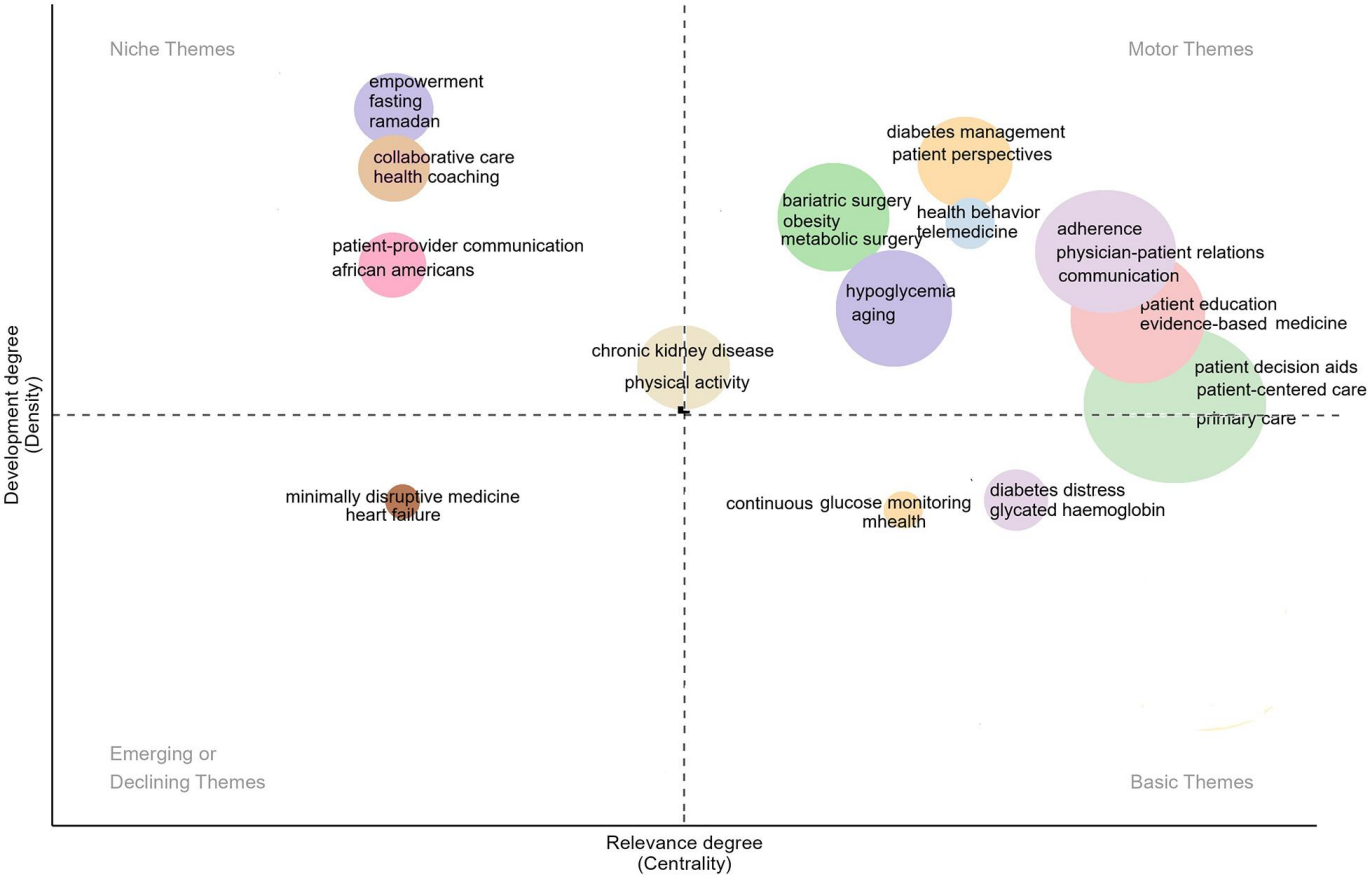
**Thematic map based on author keywords using callon's centrality and density.** Each bubble represents a keyword cluster; bubble size is proportional to the number of occurrences. The *x*-axis shows centrality (connection with other themes) and the *y*-axis shows density (internal cohesion). Quadrants represent motor, basic, niche, and emerging/declining themes.

The top ten most frequently used keywords are listed in Table 6. The thematic map (Figure 6) was created from the authors’ keywords. Using the median Callon centrality (0.083) and Callon density (50.0) as cut-points, themes are categorized as follows: Motor, Basic, Niche and one Emerging/Declining cluster. On the thematic map, the largest cluster is situated at the boundary between the basic and motor themes quadrants. This cluster encompasses concepts such as patient-centered care, primary care, and patient decision aids, with patient decision aids as the most prominent theme (Cluster Frequency = 163, Centrality = 4.74, Density = 51.11). Concepts within the Basic quadrant include diabetes distress and continuous glucose monitoring. The niche quadrant encompasses topics such as fasting (specifically during Ramadan) and patient-provider communication within African American communities. Lastly, the Emerging/Declining cluster includes such problems as minimally disruptive medicine and heart failure.

**Table 6:** Top 10 author's keywords.

Words	Occurrences (n)
patient decision aids	35
patient-centered care	14
primary care	14
self-management	13
adherence	11
patient education	11
quality of life	10
physician-patient relations	8
evidence-based medicine	7
patient preferences	7

## 4 Discussion

Our bibliometric analysis examines the evolution of research on shared decision-making (SDM) in type 2 diabetes globally between 2000 and 2024. By following these developments, the study not only charts the field's growth but also highlights where further research is most needed.

### 4.1 Social structure

Over time, SDM publications have risen steadily, peaking in 2023. The United States led the way with a significant margin, followed by the United Kingdom and the Netherlands.Network analysis revealed that the United States and the United Kingdom serve as central connectors, linking diverse research regions through established collaborations and shared academic networks. Mayo Clinic leads institutional production, followed closely by Harvard University, both in the United States. Maastricht University in the Netherlands ranked third. Of the top 10 most productive institutions, six are in the United States, two in the Netherlands, while Canada and Malaysia each contribute one. These results align with Lu's bibliometric analysis (2009–2018), which also found that the United States was the leading country and the host of the most productive university (7). What stands out, however, is that no UK institutions appear in the top 10, despite the United Kingdom ranking second overall in productivity. This suggests that the UK's output is more spread across multiple centers rather than concentrated in a few dominant ones. Centrality analysis also highlights Montori V.M., who not only had the highest PageRank score but also acted as a key bridge linking different research communities. For young researchers, we recommend establishing connections with these institutions and the previously mentioned author to gain more experience in this field.

The bibliometric study demonstrated a marked underrepresentation of the Global South in SDM research for T2DM. This geographic imbalance elicits concerns about the generalizability of existing SDM models and tool, which have been developed predominantly in high-income Western contexts (3). Further research across diverse cultural and resource settings is needed to better understand how contextual factors influence the implementation and effectiveness of SDM in T2DM.

### 4.2 Intellectual structure

The co-citation network provides further insight into the intellectual landscape. The most frequently co-cited references were studies such as Mullan R.J. (2009) (20), Weymiller A.J. (2007) (21), Branda M. (2013) (23), and Mann D.M. (2010) (22). These references have become cornerstones for later work, including the development of one of the first patients’ decision aids for antihyperglycemic agents and statin selection: the Diabetes Medication Choice and Statin Medication Choice Decision Aids by Mayo Clinic. Stacey D (2017) (24) published a systematic review regarding decision aids for multiple conditions, including T2DM. On the other hand, Turner (1998) (28) focuses on the clinical outcomes of T2DM. The co-citation network also highlights landmark publications related to the SDM conceptual framework. Charles (1997) (25) framed SDM as a process that brings physician and patient together through information exchange, consensus-building, and agreement on treatment, while noting both its benefits and its challenges. Barry (2012) (26) further developed this idea, emphasizing that patient-centered care becomes a reality only when patients are actively involved in making difficult health decisions and receive the support they need to express their values and preferences without criticism from their healthcare providers.

Unfortunately, the co-citation analysis is biased toward records in the Web of Science. To mitigate this, we also reported the ranking of globally cited documents across the whole corpus, in which Arora (2000) (19) is among the most cited references. This study, which included 2,197 patients with chronic conditions, found that people with diabetes were more likely to delegate decisions to their physicians.

Thanks to growing research on Patient-Centered Care and SDM, and to the rise of decision support systems over the past two decades, healthcare professionals have begun to involve patients more meaningfully in their care (29, 30). With this shift came a noticeable increase in patients’ willingness to take part in decisions that mattered to them. A systematic review on perceptions regarding SDM in type 2 diabetes, covering studies from 2000 to 2023, captures this evolution well. It shows that most people living with diabetes not only value SDM but also want to make choices together with their providers. The picture is more mixed on the provider side; however, only some studies report positive attitudes toward involving patients (31). The review also points to the real-world factors that make-or-break SDM: patients who feel informed and motivated are more likely to engage, while those who place blind trust in their doctors or struggle with their health often step back. Likewise, providers who share information openly and communicate well create space for SDM, whereas paternalistic approaches shut it down.

### 4.3 Conceptual structure

Examining the thematic map helps us understand what's truly driving research on shared decision-making in T2DM. Motor themes are mature, highly relevant topics that drive the field forward. These are well-established, interconnected, and supported by strong evidence bases, often forming the “engine” of research agendas. They attract funding and citations. The largest cluster in the motor themes quadrant indicates that much of the focus has been on developing tools that help patients understand their care options, weigh the risks, and make decisions that feel right for them. Another motor cluster emphasizes translating scientific evidence into patient education to motivate patient participation in health decisions. The presence of terms such as “adherence,” “physician-patient relations”, and “communication” shows how the quality of interactions between physicians and patients might influence whether patients follow treatment recommendations. A qualitative systematic review supports these findings, highlighting that perceived support from healthcare providers plays a crucial role in encouraging medication adherence, primarily through honest conversations and clear explanations of how medications work (32). The co-occurrence of terms such as “diabetes management” and “patient perspectives” indicates a body of literature where the patient perspective directly impacts clinical decision-making, such as insulin initiation (33–35), choice of antidiabetic medication (36), and diet (37). Additional themes in the motor quadrant include hypoglycemia — one of the most feared adverse events of treatment — and aging, which increases the risk of hypoglycemia. Collectively, this cluster reflects research focused on balancing glycemic control with patient safety. SDM is especially important here, as treatment goals in older adults often require individualization, consideration of comorbidities, and deintensification (38–40). We can also see that bariatric surgery is no longer a side story in obesity and diabetes care; it's moving to the center stage. Studies show it often leads to bigger, longer-lasting weight loss and more frequent diabetes remission than lifestyle changes alone (41). But surgery also carries real risks, making shared decisionsessential. Tools that predict outcomes and consider each patient's history — such as body-mass index (BMI), joint pain, or past struggles — help match the right person to the right path (42, 43). What was once a specialized focus is becoming a core theme: better results, yes, but also a deeper partnership between patients and clinicians in deciding the way forward.

Ramadan-focused themes such as fasting and empowerment appear as Niche Themes: specialized, high internal cohesion, but a lower connectivity with other topics. Empowerment-based, shared decision-making during Ramadan has been shown to improve glycemic control safely and foster lasting behavioral change (44, 45). Far from peripheral, these approaches offer a scalable way to support high-risk Muslim patients who choose to fast—underscoring the gap between how research is clustered and how it impacts real lives. Patient-provider communication and African Americans as a Niche Theme reveal that African Americans with diabetes experience systemic disparities in SDM due to issues like mistrust, a perceived racial power imbalance, and low-quality communication (46). Paradoxically, quantitative data show these patients are just as willing to engage in SDM and are more likely to initiate discussions about their care proactively than white people (47). This is a critical area that requires physician training, cultural competence, and systemic interventions to bridge the gap between patient willingness and real-world clinical practice.

We also highlight a transitional theme focusing on managing chronic kidney disease (CKD) in patients with T2DM. Nee et al. (48) identified barriers to optimal care, including low CKD awareness, suboptimal education, clinical inertia, lack of patient engagement, and high medication costs. They recommend shared decision-making (SDM) within a Chronic Care Model framework to address these barriers and improve patient outcomes. Additionally, another article presents the perspectives of two patients with T2DM and CKD who are part of the KDIGO Working Group (49). Their insights encourage other patients to ask questions and voice their concerns when making healthcare decisions with their physicians. Conversely, clinicians are also encouraged to inquire more about patients’ concerns and preferences to develop personalized care plans tailored to individual needs. “Health coaching” and “collaborative care” are also positioned within the niche themes quadrant, growing scholarly attention to patient empowerment, multidisciplinary coordination, and behavioral interventions as complementary strategies to traditional biomedical models in diabetes. These themes emphasize patient self-management, shared decision-making, and integrated care delivery—approaches with strong potential to enhance outcomes but that remain relatively peripheral in mainstream clinical practice.

The Basic Themes quadrant includes broad, foundational topics that are highly relevant but may be underdeveloped or fragmented. In our analysis, we identify themes such as “continuous glucose monitoring” and “e-health” within a single cluster. Studies in this cluster include the POWER2M trial, which examines an e-health system supporting diabetes self-management and employs explicit SDM strategies (50). Other studies in this cluster involve using continuous glucose monitoring, a tool that enhances SDM through data-driven goals. Additional themes under this quadrant include “diabetes distress” and “glycated hemoglobin”. Diabetes distress relates to frustration with self-care demands, anxiety about the future, concerns about the quality and cost of care, and perceived lack of support from family and friends. This process can contribute to worsening glycemic control (51). SDM might reduce distress by eliciting patients’ values and treatment preferences (52).

Lastly, the Emerging/Declining themes are considered peripheral, immature topics with weak connections and cohesion. These could be nascent trends (emerging) or fading ones (declining), often exploratory. In our case, we find themes such as “minimally disruptive medicine”, which is a plan of care, jointly developed by patients and clinicians, that prioritizes patient needs while minimizing treatment burden (53). This approach is essential for patients with T2DM, as these patients often have comorbidities that require their own treatment (54).

### 4.4 Strengths and limitations

Our study is the only one published to date using a bibliometric review methodology, allowing for an in-depth understanding of information relevant to researchers and clinicians regarding the characteristics of articles published up to 2024. In addition, this review was conducted following the highest quality standards and using tools specifically suited for this type of study, such as *bibliometrix* (R), which enables the generation of a comprehensive report. Despite the fact that our study draws on three major databases (Scopus, Web of Science, and PubMed), these sources tend to present mostly English-language articles. Thus, we may have overlooked contributions from non-English-speaking countries, which could impact the representation of collaboration networks and lead to an underestimate of international partnerships.

Because citations take time to accumulate, newer studies often don't receive immediate recognition. As a result, citation counts can underestimate the current influence or importance of recently published work, making it challenging to capture the actual, up-to-date impact of emerging research.

This bibliometric review provides a snapshot of the development of shared decision-making in type 2 diabetes. It highlights the key researchers, leading journals, and most active countries, helping us see where the field currently stands. More importantly, the findings can inform academia and clinical practice by highlighting the importance of involving patients in decisions about their care.

### 4.5 Implications for policy and practice

To remedy these gaps and impove the global relevance of SDM in T2DM care, the following targeted actions are recommended:

- Researchers: emphasize equitable international collaborations and adopt participatory approaches, such as co-designing SM tootl with communities in low-and middle-income countries to better incorporate local values.
- Clinicians and tool developers: adapt and validate decision aids in culturally diverse and resource-constrained settings
- Funding agencies: incentivize inclusive research through dedicated grants and mechanisms that encourage North-South partnerships and capacity building.
- Journals: Promote diversity by publishing a special issue on SDM in neglected regions and actively encouraging submissions from low-and middle-income countries.

## 5 Conclusions

This bibliometric analysis of shared decision-making (SDM) in type 2 diabetes mellitus (T2DM) from 2000 to 2024 reveals a field with a steady annual growth rate, led by Anglo-European hubs such as the Mayo Clinic and centered on motor themes, including patient decision aids and adherence. The underrepresentation of the Global South underscores a critical gap that limits the cultural adaptability of SDM models. Addressing this requires inclusive research prioritizing equitable collaborations and capacity-building in underrepresented regions. These insights can guide clinicians and policymakers to implement SDM more effectively, fostering patient-centered T2DM care worldwide. Expanding global perspectives will ensure SDM fulfills its potential as a cornerstone of equitable, personalized diabetes management.

## Data Availability

The original contributions presented in the study are included in the article/Supplementary Material, further inquiries can be directed to the corresponding authors.
