# Supplementary material for: Mapping research trends in shared decision-making for type 2 diabetes mellitus: a bibliometric study
Source: Front Health Serv. 2026 May 28;6:1799382. doi: 10.3389/frhs.2026.1799382 (PMC13253506; doi:10.3389/frhs.2026.1799382)
Supplement: Supplementary file 2 [file Table2.docx]

**Supplementary file 1. Search Strategy**

| **Data base** | **Search Strategy** |
| --- | --- |
| **Web of Science** | TS=( (("shared decision making" OR "shared decision-making" OR "patient-centered decision making" OR "patient centred decision making" OR "informed decision making" OR "informed decision-making" OR "participatory decision making" OR "participatory decision-making" OR "joint decision making" OR "joint decision-making" OR "toma de decisiones compartidas" OR "toma de decisiones compartida" OR "herramienta decisional" OR "decisiones informadas" OR "participacion en la toma de decisiones") OR ("patient decision aid*" OR "decision aid*" NEAR/3 (patient* OR shared OR participat*))) |
|  | **AND** |
|  | (("type 2 diabetes" OR "type 2 diabetes mellitus" OR "type II diabetes" OR "type II diabetes mellitus" OR "diabetes mellitus type 2" OR "diabetes mellitus type II" OR "T2DM" OR "T2D" OR "diabetes mellitus tipo 2" OR "diabetes mellitus tipo II")) |
|  | **NOT** |
|  | ("software defined memory" OR "semantic data model*" OR "SDN" OR "SDM-IO") |
|  | **NOT** |
|  | ("physician-only" OR "clinician tool*" OR "doctor decision*" OR "provider tool*")) |
| **Scopus** | TITLE-ABS-KEY ( "shared decision making" OR "shared decision-making" OR "decision aid*" OR "patient decision aid*" OR "informed decision making" OR "participatory decision making" OR "joint decision making" OR "toma de decisiones compartida" OR "toma de decisiones en conjunto" OR "ayuda para la toma de decisiones" OR "herramienta de decisión del paciente" OR "toma de decisiones informada" OR "toma de decisiones participativa" OR "toma de decisiones conjunta") |
|  | **AND** |
|  | TITLE-ABS-KEY ( "type 2 diabetes" OR "type II diabetes" OR "T2DM" OR "diabetes mellitus type 2" OR "diabetes tipo 2" OR "diabetes tipo II" OR "T2DM" OR "diabetes mellitus tipo 2" ) |
|  | **AND** |
|  | PUBYEAR > 2000 AND PUBYEAR < 2025 AND ( LIMIT-TO ( DOCTYPE , "ar" ) OR LIMIT-TO ( DOCTYPE , "re" ) ) |
| **Pubmed** | ( "shared decision making"[Title/Abstract] OR "shared decision-making"[Title/Abstract] OR "patient decision aid*"[Title/Abstract] OR "informed decision making"[Title/Abstract] OR "participatory decision making"[Title/Abstract] OR "joint decision making"[Title/Abstract]) |
|  | **AND** |
|  | ( "type 2 diabetes"[Title/Abstract] OR "type II diabetes"[Title/Abstract] OR "T2DM"[Title/Abstract] OR "diabetes mellitus type 2"[Title/Abstract] ) |
